# Chirurgie Antiseptique

**Published:** 1876-10

**Authors:** 


*Chirurgie Antiseptique Principes Modes D'applications et Resultants du Pansement De Lister.** Par Le Dr. Just Lucas-Championniere. Paris: Baillière et Fils: 1876.

American physicians are often so hurried by the general habit of "rushing things" which is so characteristic that but few of them have given much attention to Lister's method of so called anti-septic treatment. Now that Lister has been to the pains of coming across the water to explain his method at the International Congress, it is very probable that physicians will take a little more interest in the subject. The work before us is a French exposition of the mode of Lister, and is written by one who is an enthusiast on the subject. He details quite minutely the germ theory, upon which the treatment is based, and explains all the processes and variations of the anti-septic dressing of wounds, to obtain a knowledge of which he spent some time in Edinburgh under the tuition of Lister. He gives an account of some of the most notable results obtained by this method, and urges its trial and adoption by his countrymen. The work contains all the information that could be desired, and it is presented in a style which though eminently terse is nevertheless pleasing.

